# Association of TNF-*α* with Impaired Migration Capacity of Mesenchymal Stem Cells in Patients with Systemic Lupus Erythematosus

**DOI:** 10.1155/2014/169082

**Published:** 2014-11-24

**Authors:** Linyu Geng, Xia Li, Xuebing Feng, Jiyun Zhang, Dandan Wang, Jinyun Chen, Rui Liu, Haifeng Chen, Lingyun Sun

**Affiliations:** Department of Rheumatology and Immunology, The Affiliated Drum Tower Hospital of Nanjing University Medical School, 321 Zhongshan Road, Nanjing 210008, China

## Abstract

Previous studies indicated that bone marrow mesenchymal stem cells (BMSCs) from patients with systemic lupus erythematosus (SLE) exhibited impaired capacities of proliferation, differentiation, and immune modulation. Considering that migration capacity is important for the exertion of BMSCs functions, the defects in migration might contribute to BMSCs dysfunction in SLE patients. In this study, we showed that the migration capacity of SLE BMSCs was remarkably impaired in comparison with those of healthy controls. Increased tumor necrosis factor *α* (TNF-*α*) in SLE serum significantly inhibited the migration capacity and *in vivo* homing capacity of SLE BMSCs via a specific TNF receptor I (TNFRI) manner, in which decreased HGF mRNA production caused by the activation of I kappa B kinase beta (IKK-*β*) pathway is partially involved. To our knowledge, this is the first report to discuss the possible mechanisms for impaired migration of BMSCs in SLE patients. Our results suggest that inhibition of TNF-*α* pathway might be helpful for accelerating BMSCs migration to the inflammatory microenvironment in SLE patients, thereby having a potential role in SLE treatment.

## 1. Introduction

Bone marrow-derived mesenchymal stem cells (BMSCs) are nonhematopoietic cells located in bone marrow microenvironment, characterized by their self-renewal and pluripotent differentiation capability as well as the tropism for sites of tissue damage [1]. Accumulating evidence has shown that BMSCs by intravenous infusion could migrate to the injury site to alleviate destructive inflammation and promote tissue repair through their trans-differentiation and paracrine action, which has been demonstrated in bone fracture [2], myocardial infarction [3], ischemic injuries [4], wound healings [5], and autoimmune diseases [6], making them particularly interesting candidates for therapeutic cell transplantation.

Many inflammatory mediators secreted by inflammatory microenvironment are involved in regulating BMSCs migration [7, 8], such as stromal cell derived factor-1*α* (SDF-1*α*), vascular cell adhesion molecule 1 (VCAM-1), and hepatocyte growth factor (HGF) [9]. Besides these chemokines, tumor necrosis factor *α* (TNF-*α*) has also been suggested to play a role in this process. TNF-*α* is one of the major cytokines in response to various injuries and can exert its divergent biological effects through binding with two distinct cell surface receptors, TNF receptor I (TNFRI), and TNF receptor II (TNFRII) [10]. Normally, TNF-*α* may increase BMSCs migration through the activation of nuclear factor kappa-B (NF-*κ*B) signaling pathway, during which I kappa B kinase beta (IKK-*β*) acts as the rate-limiting enzyme [11–13]. However, it has been recently illustrated that TNF-*α* could downregulate MSCs migration in animal model of diabetes mellitus [14, 15], prompting that TNF-*α* may exert different roles in MSCs migration under different status.

Systemic lupus erythematosus (SLE) is an autoimmune disease characterized by multiorgan involvements and a wide array of clinical manifestations [16]. Recently, SLE has been postulated to be a stem cell disorder disease [17]. MSCs transplantation (MSCT) has been shown to be as a powerful strategy in the treatment of refractory and severe SLE patients [18–20], suggesting that bone marrow microenvironment in SLE patients may be somewhat functionally deficient, and consistent with this, compared with autologous MSCT, our data suggest that allogeneic MSCT is more effective and safe for treating severe refractory SLE patients [20]. Moreover, our previous studies have demonstrated there were some characteristic abnormalities of BMSCs from SLE patients, for example, enhanced senescence and apoptosis [21] and abnormal gene expression profile [22]. Considering that migration capacity is important for the exertion of BMSCs functions, the defects in migration might contribute to low efficacy for autologous MSCT in treating of refractory SLE patients. However, whether there are some defects in migration of SLE BMSCs and the molecular mechanisms involved in this process remains largely unknown. In this paper, to help better understanding the characteristics of BMSCs in SLE patients, the capacity of SLE BMSC migration and the role TNF-*α* in the regulation of migration were investigated.

## 2. Materials and Methods

### 2.1. Patients and Healthy Controls

Bone marrow (BM) was obtained from 6 SLE patients (mean age of 38 ± 4 years) and 6 healthy controls (HC, mean age of 39 ± 7 years). All SLE patients fulfilled 4 or more criteria according to the revised 1997 American College of Rheumatology criteria for SLE [23]. None of the patients was accompanied with other connective tissue diseases or had family history of SLE. All the SLE patients recruited had active diseases with the SELENA-SLEDAI (Systemic Lupus Erythematosus Disease Activity Index) scores [24] of more than 8 at the time of bone marrow aspiration. The main clinical characteristics of these patients were listed in Table 1. Serum from 20 female SLE patients (mean age of 38 ± 4 years, SLEDAI: 2~21) and 15 female healthy controls (mean age of 39 ± 7 years) was tested for TNF-*α* level. All participants were given written consent to the study approved by the Ethics Committee of the Affiliated Drum Tower Hospital of Nanjing University Medical School.

### 2.2. BMSCs Culture and Flow Cytometry (FCM)

Bone marrow mononuclear cells (MNCs) were isolated from bone marrow taken from the iliac crest of SLE patients and healthy controls by using 1.073 g/mL Ficoll separation medium (TBD, China) according to the manufacturer's protocol. MNCs were suspended at a density of 2 × 10^7^ cells per 25 cm^2^ dish in low glucose Dulbecco Modified Eagle Medium (L-DMEM) (Gibco, USA) supplemented with 10% heat inactivated fetal bovine serum (FBS) (Invitrogen, USA) and 1% antibiotic-antimycotic for adherent screening culture at 37°C in 5% (vol/vol) CO_2_. Medium containing nonadherent cells was replaced after 48 hours and then every 3 days. Cells grown up to 90% confluency were recovered by 0.25% trypsin-ethylene diamine tetraacetic acid (EDTA) (Gibco, USA) and replanted at a density of 1 × 10^6^ per 25 cm^2^.

Cells at passage 4 were analyzed by FCM and examined for morphology and adipogenic, chondrogenic, and osteogenic differentiation potential prior to the next experiments. FCM analysis showed cells were positive (>95%) for CD29, CD44, CD73, CD90, CD166, and CD105, but negative (<2%) for CD14, CD45, CD34, and HLA-DR.

10 *μ*L human TNF RI/TNFRSF1A APC-conjugated monoclonal antibody (TNF RI mAb) (R&D systems, USA) and 10 *μ*L human TNF RII/TNFRSF1B PE-conjugated monoclonal antibody (TNF RII mAb) (R&D systems, USA) were added to passage 4 BMSCs at a density of 5 × 10^6^ cells/mL in 0.1 mL PBS, respectively, incubated in the dark for 15 minutes according to the manufacturer's protocol, and analyzed by FCM to detect the expression levels of TNF RI and TNF RII on BMSCs.

### 2.3. Cell Scratch Experiment

Passage 4 BMSCs at a density of 1 × 10^5^ cells/well were seeded in six-well plates and grown up to 90% confluency. After starvation at the serum-free medium for 12 hours in the presence or absence of IKK-*β* inhibitor 2-[(aminocarbonyl)amino]-5-(4-fluorophenyl)-3-thiophenecarboxamide (TPCA-1, IC_50_ = 17.9 nM) (Chem Express, China) (40 nM) [25] and/or TNF-*α* (PeproTech, USA) (50 *μ*g/L, 100 *μ*g/L) [13, 26], straight line scratches were created with sterile tips in the cell plate, and then cells were gently washed 3 times by adding serum free L-DMEM medium. Cell movement and scratch width changes were observed at 0 and 12 hours, respectively, after the scratch experiments under an inverted microscope.

The average gaps of migration distance were measured using the edge method. Thirty points were uniformly selected at each scratch edge using the software Image-Pro Plus 6.0, the midline of which represents the scratch edge at 0 hours, and then the uniform scratch edge distance was measured 12 hours after the scratch experiment. The migration distance was calculated according to the following formula: average gaps (12 h) = edge distance (12 h) − edge distance (0 h).

### 2.4. Transwell Migration Assay

Passage 4 BMSCs at a density of 5 × 10^5^ cells/mL in 0.2 mL L-DMEM without FBS were added into the upper chamber of a 6.5 mm diameter transwell insert with a pore size of 8 *μ*m. The lower chamber in 24-well plates contained 0.5 mL of L-DMEM with 10% FBS or with 10% serum of SLE patients (incubated at 56°C for 30 min prior for complement inactivation) with the presence or absence of the anti-human TNF-*α* mAb (0.1 *μ*g/mL) (R&D Systems, Inc). After incubation at 37°C and 5% CO_2_ for 12 hours, the upper surface of the membrane was scraped gently to remove nonmigrating cells. Cells on the lower surface of the membrane that have migrated into the lower compartment of the chamber were then fixed in 4% paraformaldehyde for 10 minutes and stained in Giemsa solution for 3 minutes according to the manufacturer's protocol. The number of migrating cells was quantified using image pro-plus 6.0 in five random morphologic fields per well, averaged the values, and was multiplied by the ratio of per microscopic field area to bottom area per 24 wells afterward, and, finally, its proportion in total number of BMSCs (1 × 10^5^) seeded in the upper chamber was considered as the migration rate.

Passage 4 BMSCs prestimulated with the present or absent of IKK-*β* inhibitor TPCA-1 (40 nM) and/or TNF-*α* (50 *μ*g/L, 100 *μ*g/L) (PeproTech, USA) in serum-free medium for 2 hours were also cultured in transwell system as mentioned above. And so did the transwell assay for passage 4 BMSCs pretreated with recombinant human TNF-*α* (50 *μ*g/L, 100 *μ*g/L) with or without human TNF RI/TNFRSF1A mAb (R&D systems, USA) and human TNF RII/TNFRSF1B mAb (R&D systems, USA) in serum-free medium for 2 hours.

### 2.5. Cell Counting Kit-8 (CCK-8) Assay

Passage 4 BMSCs at a density of 1 × 10^5^ cells/mL were seeded in 96-well plates with 0.1 mL L-DMEM (10% FBS) and incubated for 24 hours after application of CCK-8 kit, and values of OD450 for each group were read by the microplate reader according to manufacturer's protocol.

### 2.6. Enzyme-Linked Immunosorbent Assay (ELISA) Analysis

Serum from 20 SLE patients and 15 healthy controls was collected and stored at −20°C until assayed. The concentrations of TNF-*α* of each sample were measured using commercial ELISA kit (R&D Systems, Inc.) according to the manufacturer's protocol. The optical density (OD) was measured in a detector (Bio-Rad, USA) at 450 nm wavelength.

### 2.7. Quantitative Real-Time Polymerase Chain Reaction (RT-PCR) Analysis

Passage 4 BMSCs from SLE patients were cultured in a 6-well plate (2.0 × 10^5^ viable cells). Total cellular RNA was extracted using Trizol reagent (Invitrogen, USA) and stored at −70°C. For quantitative RT-PCR, single-stranded cDNA was synthesized in a 200 *μ*L reaction volume containing 30 *μ*L total RNA using PrimerScript RT reagent Kit (TaKaRa, China). Quantitative RT-PCR was performed on an ABI 7500 FAST real-time PCR detection system (Applied Biosystems, USA) using SYBR Green detection mix (Takara, China). StepOne Software v2.1 was used to detect the mix, and the thermal profile for RT-PCR was 95°C for 30 seconds, followed by 40 cycles of 5 seconds at 95°C with 34 seconds at 60°C. The specific primers (TaKaRa, China) used are shown in Table 2.

Relative expression of the target genes was calculated with the 2^−ΔΔCt^ method. Briefly, a value for the cycle threshold (Ct) was determined for each sample and defined as the mean cycle at which the fluorescence curve reached an arbitrary threshold. The ΔCt for each sample was then calculated according to the formula Ct target gene − Ct GAPDH; ΔΔCt values were then obtained by subtracting the ΔCt of a reference sample (average ΔCt of the control group) from the ΔCt of the studied samples. Finally, the expression levels of the target genes in the studied samples as compared with the reference sample were calculated as 2^−ΔΔCt^.

### 2.8. Western Blot Analysis

2 × 10^5^ viable cells precultured in 6-well plates were harvested and lysed with 80 *μ*L RIPA buffer supplemented with protease and phosphatase inhibitors for 20 minutes on ice. Cell lysates were then centrifuged at 13000 g, 4°C for 10 minutes, and the supernatant was gathered. Protein concentration was measured using the BCA method. Equal amounts of protein (20 *μ*g) were separated by SDS-PAGE (sodium dodecyl sulfate polyacrylamide gel electrophoresis) and blotted to a polyvinylidene difluoride (PVDF) membrane (Millipore, USA) before it was blocked with 5% skimmed milk for 1 hour. Overnight incubation at 4°C with rabbit anti-IKK-*β* antibody (Abcam, Britain), anti-phospho-IKK-*β* antibody (Abcam, Britain), and anti-GAPDH antibody (Abcam, Britain) was carried out before visualized with HRP-conjugated secondary antibody (Millipore, USA) at room temperature for 1 hour followed by detection with FluorChem FC2 System (Alpha Innotech Corporation, USA).

### 2.9. *In Vivo* Homing Assay

Breeding C57BL/6 female mice were purchased from Model Animal Research Center of Nanjing University. All mice were fed with standard chow diet through all age and maintained in a temperature-controlled room with a 12 h light/dark cycle according to the approved protocol by the Affiliated Drum Tower Hospital of Nanjing University Medical School Committee on use and care of experimental animals.

To determine the role of TNF-*α* in* in vivo* homing ability of SLE BMSCs, passage 4 SLE BMSCs at a density of 5 × 10^6^ cells/mL in 0.2 mL PBS were pretreated with recombinant human TNF-*α* (50 *μ*g/L, 100 *μ*g/L) in the presence or absence of specific neutralizing antibodies to TNFRI and TNFII for 24 hours, and then cells were labeled with 5(6)-carboxyfluorescein diacetate succinimidyl ester (CFSE) (Takara, China) according to the manufacturer's protocol and infused in MRL/lpr mice at 16 weeks of age intravenously. Labeling efficacy of CFSE-labeled BMSCs was >98%. For stem cell engraftment and histological studies, mice were sacrificed 24, 48, and 72 hours after injection, and organs including heart, liver, spleen, lung, kidney, and lymph nodes were collected and wrapped in dark at −70°C. Quantification of BMSCs engraftment in organs tissue samples was fixed in 4% paraformaldehyde (Electron Microscopy Sciences, PA), embedded in 30% sucrose/PBS and in Tissue-Tek OCT Compound (Sakura Finetek, CA). Fifteen sections per organ were analyzed by fluorescent microscopy using a fluorescent inverted microscope Axio observer A1 (ZEISS, Germany), images were acquired with an objective magnification of ×10 (×40 total magnification) using an Olympus DP30BW camera (Olympus, Japan), and the mean fluorescence intensity of CFSE + SLE BMSCs was observed in five to six random fields.

### 2.10. Statistical Analysis

All results were presented as mean ± S.E.M. of data from at least three separate experiments. Differences between two groups were determined by unpaired Student's *t*-test if the variance was normally distributed. Comparisons among three or more groups were conducted using one-way ANOVA. Statistical analysis was performed using GraphPad Prism 5 software and a value of *P* < 0.05 was considered statistically significant.

## 3. Results

### 3.1. Decreased Migration Capacity of SLE BMSCs

**Figure 1:**
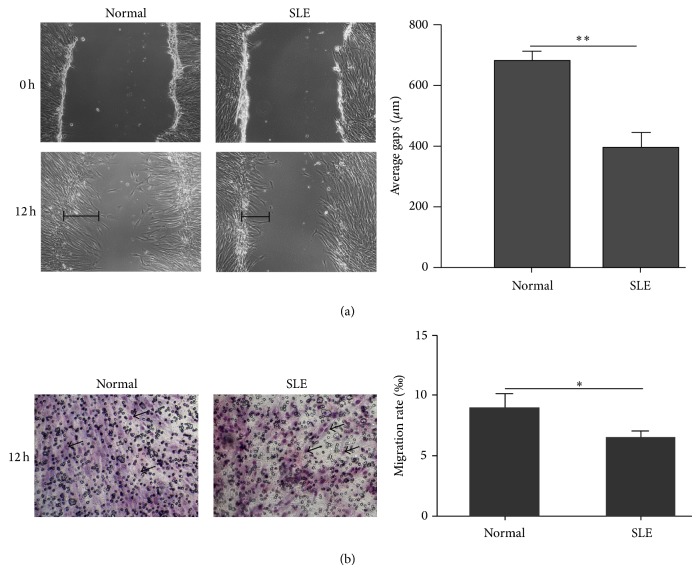
Migration capacity was significantly decreased in BMSCs from SLE patients. (a) The scratches healed faster in BMSCs from healthy controls than that from SLE patients 12 hours after the scratch experiments. The length of average healed gaps was illustrated by the black line. (b) The migration rate of BMSCs from SLE patients was remarkably reduced compared with that of healthy controls by transwell migration assay. Migrated BMSCs stained in Giemsa solution (marked by black arrows in the figure) were quantified by image pro-plus 6.0 software to obtain the migration rate per morphologic field randomly selected. ^*^ *P* < 0.05, ^**^ *P* < 0.01.

To compare the migration capacity of BMSCs from healthy controls and SLE patients, cell scratch and transwell assays were performed. We found that, 12 hours after the scratch experiments, BMSCs from SLE patients healed slower as shown by shorter healed gaps than that from healthy controls (384.8824 ± 51.8 *μ*m versus 673.5000 ± 34.4 *μ*m, *n* = 6, *P* < 0.01) (Figure 1(a)). Consistently, transwell assay also revealed that the migration rate of SLE BMSCs was remarkably reduced compared with that of healthy controls (6.470 ± 1.390‰ versus 8.914 ± 2.920‰, *n* = 6, *P* < 0.05) (Figure 1(b)), confirming that the migration capacity of SLE BMSCs was weakened* in vitro*. BMSCs from patients with high SLDAI score (*r* = −0.54) (Supplementary Figure  1(a) available online at http://dx.doi.org/10.1155/2014/169082), high proteinuria (*r* = −0.70) (Supplementary Figure  1(b)), or taking high doses of steroids (*r* = −0.54) (Supplementary Figure  2(a)) as well as hydroxychloroquine (HCQ) (*r* = −0.77) (Supplementary Figure  1(b)) tended to have decreased migration rate in transwell assay, yet none of them reached the significance.

### 3.2. TNF-*α* Inhibited the Migration of SLE BMSCs

**Figure 2:**
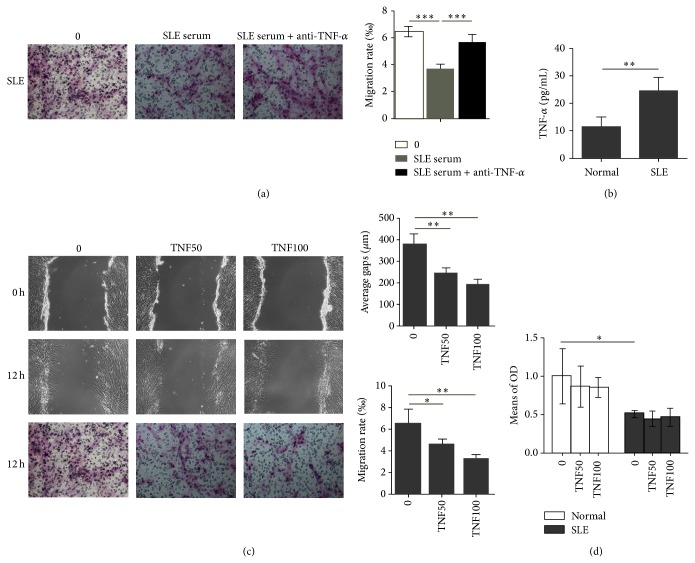
Association of elevated serum TNF-*α* with impaired migration of SLE BMSCs. (a) TNF-*α* blockers restored the decreased SLE BMSCs migration capacity induced by SLE serum (*n* = 6). (b) Serum level of TNF-*α* was significantly elevated in SLE patients compared to that of healthy controls. (c) TNF-*α* significantly decreased migration capacity of SLE BMSCs both in the wound healing (*P* = 0.003) and transwell migration assays (*n* = 6). (d) TNF-*α* at either 50 *μ*g/L or 100 *μ*g/L had no impact on the proliferation rate of BMSCs from both healthy controls and SLE patients. TNF50: TNF-*α*: 50 *μ*g/L; TNF100: TNF-*α*: 100 *μ*g/L. ^*^ *P* < 0.05; ^**^ *P* < 0.01.

To explore the factors involved in the regulation of migration, transwell assay was applied to detect the migration capacity of SLE BMSCs in medium containing 10% SLE serum with or without anti-human TNF-*α* mAb. Interestingly, our data showed that the migration capacity of SLE BMSCs was further inhibited by SLE serum (3.6765 ± 0.3765‰ versus 6.470 ± 1.390‰, *n* = 6, *P* < 0.001), and the effect was diminished after TNF-*α* blocking (5.6647 ± 0.5700‰ versus 6.470 ± 1.390‰, *n* = 6, *P* < 0.001) (Figure 2(a)). Next, we measured serum level of TNF-*α* in SLE patients. Compared with healthy controls, serum level of TNF-*α* was significantly elevated in SLE patients (24.288 ± 4.995 pg/mL versus 11.523 ± 3.155 pg/mL, *n* = 6, *P* < 0.01) (Figure 2(b)). To confirm a direct role of TNF-*α* on migration of SLE BMSCs, recombinant human TNF-*α* (50 *μ*g/L, 100 *μ*g/L) was added to the cultures, and significant decrease in average healed gaps of SLE BMSCs (247.1765 ± 27.1765 *μ*m versus 190.7647 ± 22.5700 *μ*m, *n* = 6), and cell migration rate (4.56 ± 0.51‰ versus 3.24 ± 0.37‰, *n* = 6) (Figure 2(c)) was detected as compared to those without TNF-*α* treatment, suggesting that TNF-*α* is an important antagonist for SLE BMSCs migration. Lower concentrations of TNF-*α* (1 ng/L, 0.1 *μ*g/L, and 1 *μ*g/L) (6.04 ± 0.12‰ versus 5.53 ± 0.30‰ versus 5.08 ± 0.18‰, *n* = 6) could not efficiently alter the migration rate of SLE BMSCs and a higher concentration of TNF-*α* (1000 *μ*g/L) (3.23 ± 0.21‰, *n* = 6) did not alter the migration rate of SLE BMSCs significantly compared to TNF-*α* at 50 *μ*g/L (4.56 ± 0.51‰, *n* = 6) and at 100 *μ*g/L (3.24 ± 0.37‰, *n* = 6), indicating that TNF-*α* induced the inhibition of SLE BMSCs migration in a nondose-dependent manner (data not shown).

To find out whether TNF-*α* inhibited SLE BMSCs migration through the negative regulation of cell growth rate, the proliferation rate of BMSCs was measured by CCK-8 assay. Our data showed that BMSCs proliferation in SLE patients was significantly lower than that in healthy controls (0.51 ± 0.05 versus 1.20 ± 0.35, *n* = 6, *P* < 0.01), while TNF-*α* at either 50 *μ*g/L or 100 *μ*g/L had no significant impact on the proliferation rate of BMSCs from both healthy controls and SLE patients (Figure 2(d)).

### 3.3. TNF-*α* Decreased Migration and* In Vivo* Homing Capacity of SLE BMSCs via TNFRI

**Figure 3:**
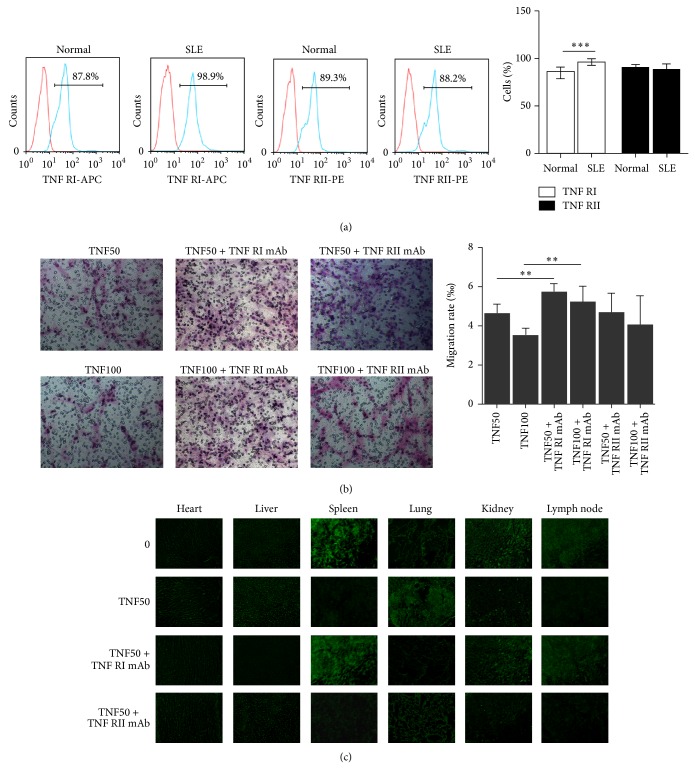
TNF-*α* decreased migration and* in vivo* homing capacity of SLE BMSCs via TNFRI. (a) TNFRI percentage was increased in SLE BMSCs (*n* = 4). (b) Ablation of TNFRI significantly converted TNF-*α* induced impaired migration capacity SLE BMSCs. (c) Compared with PBS treated SLE BMSCs group,* in vivo* homing capacity of TNF-*α* (50 *μ*g/L) pretreated SLE BMSCs was markedly impaired, and preincubation of BMSCs with TNF-*α* together with TNFRI mAb instead of TNFRII mAb converted SLE BMSCs homing capacity with more SLE BMSCs homing for kidney, lymph node, and spleen. TNF50: TNF-*α*: 50 *μ*g/L; TNF100: TNF-*α*: 100 *μ*g/L. ^**^ *P* < 0.01; ^***^ *P* < 0.001.

TNFRI and TNFRII largely play discrete roles in mediating functions of TNF-*α*. To find out which subtypes mediated the effects of TNF-*α* on BMSCs, levels of TNFRI and TNFII on BMSCs from SLE patients and healthy controls were detected by FCM. Compared to healthy controls, TNFRI percentage was increased in SLE BMSCs (84.4 ± 4.8% versus 95.6 ± 2.2%, *n* = 4, *P* < 0.001) (Figure 3(a)). Ablation of TNFRI but not TNFRII significantly converted TNF-*α* induced impaired migration capacity SLE BMSCs (Figure 3(b)), supporting that TNF-*α* induced impaired migration of SLE BMSCs was mediated by TNFRI instead of TNFRII.

To further validate the hypothesis,* in vivo* homing capacity of BMSCs was measured. Significantly impaired* in vivo* homing capacity of SLE BMSCs was observed after TNF-*α* (50 *μ*g/L) treatment, by showing massively trapped MSCs within the lungs rather than inflammatory organs including kidney, lymph node, and spleen (Figure 3(c)). Preincubation with TNFRI neutralizing mAb but not TNFRII neutralizing mAb could convert TNF-*α* induced disturbance of SLE BMSCs with more SLE BMSCs homing to kidney, lymph node, and spleen (Figure 3(c)). Similar effect was detected in SLE BMSCs pretreated with 50 *μ*g/L TNF-*α* at 48 and 72 hours and 100 *μ*g/L TNF-*α* at 24, 48, and 72 hours (data not shown).

### 3.4. IKK-*β* Was Involved in TNF-*α* Impaired Migration of SLE BMSCs

**Figure 4:**
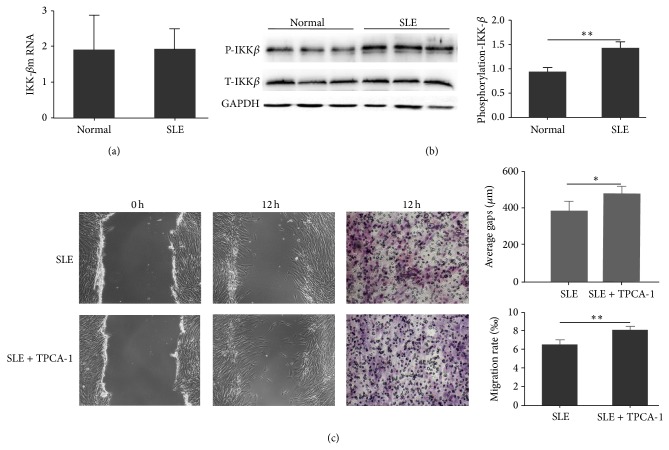
Effect of IKK-*β* in impaired migration capacity of SLE BMSCs. (a) No difference was observed in IKK-*β* mRNA level between SLE patients and normal controls. (b) Phosphorylated-IKK-*β* protein expression of SLE BMSCs (1.38 ± 0.12) was significantly increased. (c) TPCA-1, a selective inhibitor of IKK-*β*, significantly increased the migration rate of SLE BMSCs both in the wound healing (*P* = 0.03) and transwell migration assays. ^*^ *P* < 0.05; ^**^ *P* < 0.01.

Since IKK-*β* acts as the rate-limiting step in TNF-*α*-TNFRI mediated effects [11, 12], the IKK-*β* mRNA and protein levels in BMSCs from healthy controls and SLE patients were measured. There was no significant difference in IKK-*β* mRNA (Figure 4(a)) and total IKK-*β* (t-IKK-*β*) protein levels (Figure 4(b)) between SLE patients and healthy controls; however, western blot showed that the phosphorylated-IKK-*β* (p-IKK-*β*) protein level in SLE BMSCs was significantly increased compared with that from healthy controls (Figure 4(b)). To validate the role of IKK-*β* in the impaired migration capacity of SLE BMSCs, TPCA-1, a selective inhibitor of human IKK-*β*, was added into the culture medium at the concentration of 40 nM. Our results showed that the presence of TPCA-1 significantly increased the migration capacity of BMSCs from SLE patients both in the cell scratch (384.8824 ± 51.8000 *μ*m versus 466.7647 ± 41.4000 *μ*m, *n* = 6, *P* = 0.03) and transwell migration assays (6.47 ± 1.39‰ versus 8.02 ± 1.00‰, *n* = 6, *P* = 0.003) (Figure 4(c)), indicating that IKK-*β* activation participated in impaired migration capacity of SLE BMSCs.

**Figure 5:**
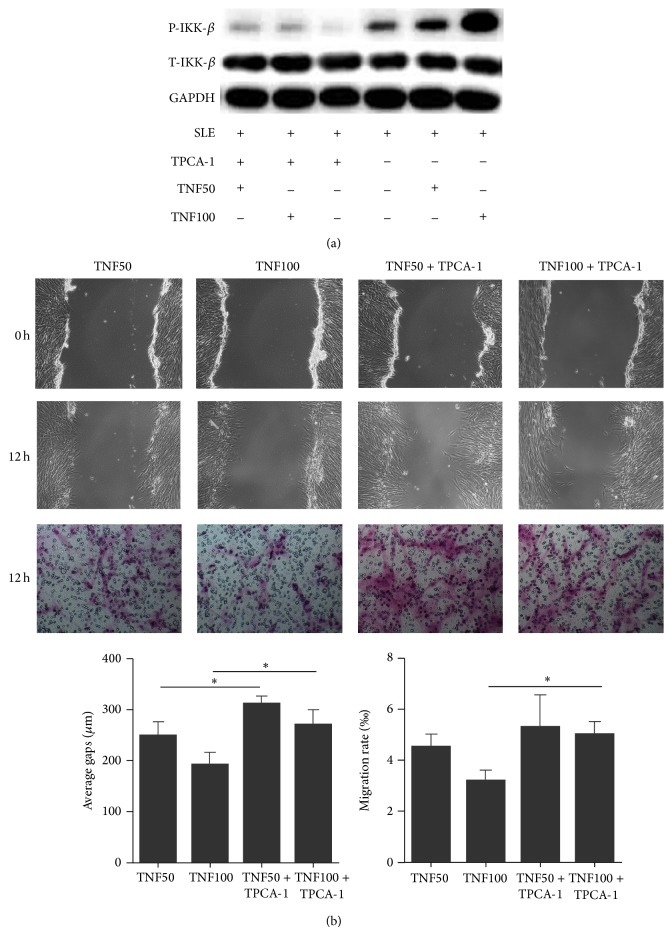
Upregulation of p-IKK-*β* involved in TNF-*α* induced abnormal migration of SLE BMSCs. (a) Protein levels of p-IKK-*β* and t-IKK-*β* in SLE BMSCs after TPCA-1 or TNF-*α* treatment. (b) TPCA-1 could reverse the effect of TNF-*α* on migration capacity of SLE BMSCs in the wound healing and transwell migration assays (*n* = 6). TNF50: TNF-*α*: 50 *μ*g/L; TNF100: TNF-*α*: 100 *μ*g/L. ^*^ *P* < 0.05.

To elucidate whether TNF-*α* induced impaired migration capacity of SLE BMSCs was mediated by the activation of IKK-*β* signaling, p-IKK-*β* level and migration capacity of SLE BMSCs were measured after culturing in the absence or presence of recombinant human TNF-*α* together with or without TPCA-1. After pretreated with TNF-*α* in serum free medium for 2 hours, markedly increased p-IKK-*β* in SLE BMSCs were detected (Figure 5(a)). Cell scratch and transwell migration assays showed that the decreased migration capacity of SLE BMSCs induced by both low (50 *μ*g/L) or high concentration (100 *μ*g/L) of TNF-*α* was all restored by TPCA-1 (Figure 5(b)), indicating that TNF-*α* induced impaired migration of SLE BMSCs could be mediated at least partially through the upregulation of p-IKK-*β*.

### 3.5. TNF-*α* and IKK-*β* Regulated SLE BMSCs Migration through the Inhibition of HGF Production

**Figure 6:**
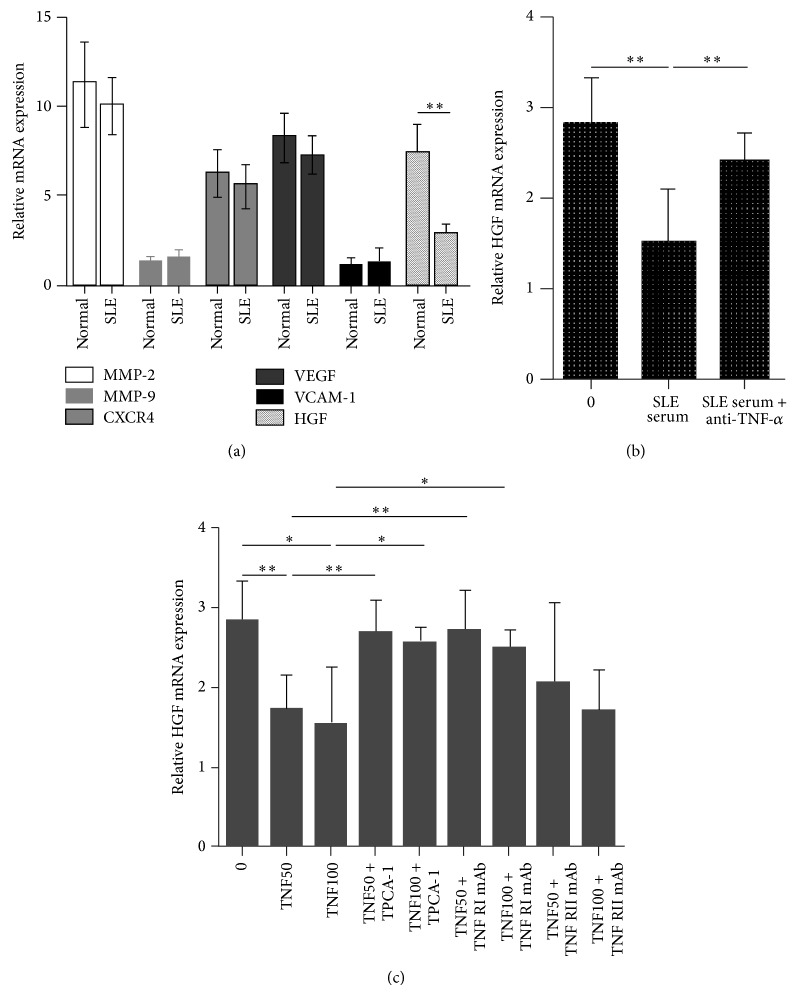
TNF-*α* and IKK-*β* regulated SLE BMSCs migration through the inhibition of HGF production. (a) Level of HGF mRNA, instead of MMP-2, MMP-9, CXCR4, VEGF, and VCAM-1, was decreased in SLE BMSCs compared with normal BMSCs. (b) HGF expression in SLE BMSCs was downregulated by SLE serum, which was abrogated with the addition of anti-TNF-*α* mAb. (c) HGF mRNA level in SLE BMSCs pretreated with recombinant human TNF-*α* with the presence or absence of anti-TNFRI mAb, anti-TNFRII mAb, or TPCA-1 (*n* = 3). TNF50: TNF-*α*: 50 *μ*g/L; TNF100: TNF-*α*: 100 *μ*g/L. ^*^ *P* < 0.05; ^**^ *P* < 0.01.

To determine which chemokines produced by BMSCs mediated the effects of TNF-*α*, mRNA levels of matrix metalloproteinase 2 (MMP-2), MMP-9, C-X-C chemokine receptor type 4 (CXCR4), vascular endothelial growth factor (VEGF), VCAM-1, and HGF in BMSCs from SLE patients and normal controls were detected by RT-PCR, and only HGF mRNA level was decreased in SLE BMSCs (Figure 6(a)). HGF expression in SLE BMSCs was inhibited by SLE serum, and the effect of SLE serum on HGF expression was abrogated with the addition of anti-TNF-*α* mAb (Figure 6(b)). Additionally, HGF expression in SLE BMSCs could also be inhibited by recombinant TNF-*α*, which was diminished in the presence of TPCA-1 or TNFRI neutralizing mAb (Figure 6(c)), suggesting that TNF-*α* and IKK-*β* acted through the inhibition of HGF mRNA production to participate in impaired migration capacity of SLE BMSCs.

## 4. Discussion

MSCs have attracted much attention among their great potential in clinical applications, making it increasingly important figuring out how these cells migrate into damaged inflammatory tissues [27]. In this study we showed for the first time that the migration capacity of BMSCs from SLE patients was decreased compared with that of healthy controls. TNF-*α* at different concentrations significantly downregulated the migration capacity of SLE BMSCs, while anti-TNF-*α* mAb ameliorated SLE serum induced inhibition of SLE BMSCs migration, suggesting the elevation of TNF-*α* in SLE patients could be involved in this process, which was quite contrary to the result in BMSCs from healthy controls [11–13]. Compared to healthy controls, TNFRI percentage was increased in SLE BMSCs, and preincubation of SLE BMSCs with TNF-*α* together with TNFRI neutralizing mAb instead of TNFRII neutralizing mAb significantly converted TNF-*α* induced impaired SLE BMSCs migration and* in vivo* homing capacity. Further, the IKK-*β* inhibitor could partially convert the effect of TNF-*α*, supporting a role of p-IKK-*β* in TNF-*α* regulated BMSCs migration in SLE patients. Level of HGF mRNA was decreased in SLE BMSCs and downregulated by SLE serum and recombinant TNF-*α*, which was diminished after TNF-*α* blocking or adding TPCA-1 to the cultures, supporting that HGF plays a role in TNF-*α* and IKK-*β* induced impairment of SLE BMSC migration. Collectively, our data indicate that the migration capacity of BMSCs is impaired in SLE patients. TNF-*α* might act through the conjunction of TNFRI to inhibit BMSCs migration, in which the activation of IKK-*β*-HGF pathway is at least partially involved.

So far, the precise molecular mechanisms of MSCs migration remain largely unknown. TNF-*α* is one of the major cytokines in response to various injuries [13, 28] and shows increased serum level in various human models of tissue injury including SLE patients [29], which may recruit BMSCs to the injury site. Several studies have illustrated that TNF-*α* could promote normal MSCs migration at the time of tissue damage such as myocardial infarction [30], tissue ischemia [4], and wound healing [5]. MSCs pretreated with 10 *μ*g/L TNF-*α* accumulated to a greater extent in the areas of ischemic damage in rat hind limbs [28], and TNF-*α* at 50 *μ*g/L significantly enhanced the migration ability of MSCs in the context of wound healing [13]. However, the role of TNF-*α* in MSCs in disease status remains controversial as studies have demonstrated that toll-like receptor 7-stimulated TNF-*α* caused bone marrow damage in SLE patients [31], and impaired wound healing in animals with diabetes mellitus has been reported to be mediated by abnormal MSCs migration response after TNF-*α* stimulation [14, 15]. In this study, our results confirmed that the serum level of TNF-*α* was significantly elevated in SLE patients, which may account for the impaired migration capacity of BMSCs in SLE.

TNFRI and TNFRII play discrete roles in mediating functions of TNF-*α*. Recent evidence has indicated that ablation of TNFRI in stem cells increases VEGF secretion, whereas the absence of TNFRII decreases VEGF production in MSCs [32], suggesting that TNFRI signaling may play a detrimental role in MSCs migration capacity while TNFRII may favor stem cell function. In our experiment, compared to healthy controls, the percentage of TNFRI was increased in SLE BMSCs, while preincubation of BMSCs with TNF-*α* together with TNFRI neutralizing mAb instead of TNFRII neutralizing mAb significantly restored the capacity of SLE BMSCs migrating and* in vivo* homing, demonstrating that TNF-*α* decreased SLE BMSCs migration via a specific TNFRI manner.

Many studies have indicated that TNF-*α* activated IKK-*β*, a key regulatory enzyme of the NF-*κ*B signal transduction pathway, plays a functional role in BMSCs invasion and migration [11, 12, 33], and blocking the activation of NF-*κ*B induced by TNF-*α* could inhibit MSCs migration and invasion [34]. Normally, NF-*κ*B is held in an inactive form in the cytoplasm by inhibitory I*κ*B proteins [35]. Upon stimulation with TNF-*α* [36], I*κ*B can be phosphorylated by IKK-*β* and subsequently inducing its ubiquination and proteosome mediated degradation, allowing nuclear translocation of NF-*κ*B and alteration of its target genes expression, such as intercellular adhesion molecule-1 (ICAM-1), VCAM-1, and HGF, and thus played a crucial role in the course of tissue injury and immune response. In this experiment, we confirmed that TNF-*α* induced impairment of SLE BMSCs migration required the activation of p-IKK-*β*, while IKK-*β* inhibitor could partially convert the effect of TNF-*α*.

Clinically, impaired migration capacity of SLE BMSCs upon TNF-*α* pretreatment may result in the slow reaction of BMSCs to the inflammatory microenvironment, thus playing a role in the pathogenesis of this disease. Our data showed that TNF-*α* pretreatment further impaired the* in vivo* homing capacity of SLE BMSCs, indicating that inhibition of the TNF-*α* pathway may accelerate BMSCs migration to the inflammatory microenvironment in SLE patients, and thereby may have a potential role in the treatment of SLE.

Chemokine receptors are thought to be involved in regulating BMSCs migrate to injury sites through interaction with chemokines and cytokines secreted by inflammatory microenvironment. It has been reported that TNF-*α* could stimulate the production of HGF to regulate human MSCs migration, which was dependent on the p38 mitogen-activated-protein-kinase (MAPK) and phosphoinositide-3-kinase (PI3K)/Akt but not c-JNK and extracellular signal-regulated kinase (ERK) signaling pathways [31]. Our results demonstrated that level of HGF mRNA was decreased in SLE BMSCs and were inhibited by both SLE serum and recombinant TNF-*α*, while the effect was diminished after TNF-*α* blocking or the presence of TPCA-1, indicating that TNF-*α* induced impaired migration of SLE BMSCs might be related with inhibition of HGF mRNA production.

Some medications, such as glucocorticoid (GC), and immunosupressors including cyclophosphamide (CYC), hydroxychloroquine (HCQ), and mycophenolate mofetil (MMF) are used extensively for the treatment of SLE. It is reported that GC increased cell adhesion to the extracellular matrix via *α*-*β*1 integrin, and thereby antagonized epidermal growth factor (EGF) induced human gastric carcinoma cells migration [37]; MMF impaired transendothelial migration of allogeneic CD4 and CD8 T cells [38], inhibited intimal hyperplasia, and attenuates the expression of genes favoring smooth muscle cell proliferation and migration [39], while granulocyte precursors accumulate in the BM during mobilization induced by CYC leading to the accumulation of active neutrophil proteases [40]. In this study, we observed trend associations between BMSCs migration and the dose of GC as well as HCQ (Supplementary Figure 2). However, since patients taking high doses of GC and HCQ were the same as those with high disease activities, the results may actually be influenced by disease activity instead of the treatments. To further clarify whether these medications and their dose would influence the migration capacity of BMSCs, more SLE patients with various disease activities are needed to be recruited.

There are also several limitations in this study. First, other cytokines including tumor necrosis factor-related apoptosis-inducing ligand (TNFRAIL) [41], interferon-*γ* (IFN-*γ*) [42], platelet-derived growth factor (PDGF) bb, and interlukin-6 (IL-6) [43] had been reported to be potent chemotactic factors for MSCs migration. It is currently unknown whether these cytokines could act synergistically with TNF-*α* to induce the impairment of SLE BMSCs migration. Second, the presence of TPCA-1 did not fully restore the migration capacity of BMSCs in our experiment, suggesting that other pathways may be involved in TNF-*α* mediated signal. Third, the effect of TNF-*α* on BMSCs migration* in vivo* has not been investigated.

Taken together, here we report that BMSCs from SLE patients had decreased migration capacity, which was related to elevated TNF-*α* serum level and was acted via a specific TNFRI manner to activate IKK-*β* and consequently inhibit HGF mRNA production. Our results may explain the low autologous MSCT efficacy for severe refractory SLE patients [20], and inhibition of TNF-*α* pathway could promote BMSCs migration to the inflammatory microenvironment in SLE patients, thereby having a potential role in SLE treatment.

## 5. Conclusions

Migration capacity of BMSCs is impaired in SLE patients, which is tightly associated with the elevation of TNF-*α* level. TNF-*α* might act through the conjunction of TNFRI instead of TNFRII to inhibit BMSCs migration, in which the activation of IKK-*β*-HGF pathway is at least partially involved.

## Supplementary Material

Supplementary Figure 1: Associations of the migration rate with clinical manifestations: a. SLEDAI score, b. white blood cells, c. hemoglobin, d. platelet, e. urine protein, f. serum creatinine, g. complement 3, h. complement 4, i. ESR, j. arthralgia, k. febrile, l. vasculitis, m. pneumonia, n. serositis.
Supplementary Figure 2: Associations of the migration rate with various treatment drugs. a, b. BMSCs from patients taking high doses of steroids (a) or HCQ (b) tended to have decreased migration rate. c,d. MMF (c) or CYC (d) treatment do not seem to have effect on SLE BMSCs migration rate.

## Figures and Tables

**Table 1:** Main clinical characteristics of SLE patients.

Patient number/age/sex	Disease duration, months	SLEDAI score	Clinical manifestations	Treatments before BM puncture
1/24/F	1	13	Febrile, cytopenia, hypohemoglobinemia, thrombocytopenia, nephritis, hypocomplementemia, ANA positive, anti-SM positive, anti-RNP positive	Pred 20 mg/day, HCQ 200 mg/day

2/45/F	216	12	Febrile, nephritis, alopecia, cutaneous vasculitis, cytopenia, hypocomplementemia hypohemoglobinemia, ANA positive, anti-dsDNA positive	Pred 10 mg/day, HCQ 400 mg/day, MMF 1.0 gm/day, CYC 0.4 gm/mo

3/56/F	120	10	Arthralgia, cutaneous vasculitis, nephritis, serositis, interstitial pneumonia, cytopenia, ANA positive	Pred 20 mg/day, HCQ 400 mg/day

4/37F	120	15	Arthralgia, cutaneous vasculitis, febrile, nephritis, cytopenia, hypocomplementemia, interstitial pneumonia, lymphadenectasis, ANA positive, anti-dsDNA positive, anti-RNP positive	Pred 30 mg/day, MMF 0.5 gm/day, CYC 0.4 gm/mo

5/16/F	8	10	Cutaneous vasculitis, arthralgia, febrile, nephritis, cytopenia, hypohemoglobinemia, ANA positive, anti-dsDNA positive, anti-SM positive, anti-RNP positive	Pred 20 mg/day, HCQ 400 mg/day

6/22/F	40	9	Nephritis, cytopenia, thrombocytopenia, serositis, ANA positive, anti-dsDNA positive	Pred 15 mg/day, CYC 0.8 gm/mo, HCQ 400 mg/day

ANA: anti-nuclear antibody, anti-dsDNA: anti-double strand DNA antibody, anti-RNP: anti-ribonuclear protein antibody, anti-SM: anti-Smith antibody, Pred: prednisone, CYC: cyclophosphamide, HCQ: hydroxychloroquine, MMF: mycophenolate mofetil; SLEDAI: SLE disease activity index; BM: bone marrow.

**Table 2:** RT-PCR primers sequences.

Gene	Upstream primer (F)	Downstream primer (R)
IKK-*β*	5′-GGCAAACCGTACTCCAAGCAC-3′	5′-CCTTGTCTGCACACTGGAGGTC-3′
CXCR4	5′-CCTCCTGCTGACTATTCCCGA-3′	5′-GGAACACAACCACCCACAAGT-3′
MMP-9	5′-AGGCCTCTACAGAGTCTTTG-3′	5′-CAGTCCAACAAGAAAGGACG-3′
HGF	5′-GAAGGTGAAGGTCGGAGTC-3′	5′-GAAGATGGTGATGGGATTTC-3′
VCAM-1	5′-CTGCACGGTCCCTAATGT-3′	5′-AAGAGCTTTCCCGGTGTC-3′
GAPDH	5′-AGAAGGCTGGGGCTCATTTG-3′	5′-AGGGGCCATCCACAGTCTTC-3′

## Abbreviations

BMSCs: Bone marrow-derived mesenchymal stem cells
SLE: Systemic lupus erythematosus
MSCT: MSCs transplantation
TNF-*α*: Tumor necrosis factor *α*
IKK-*β*: I kappa B kinase beta
p-IKK-*β*: Phosphorylated-IKK-*β*
t-IKK-*β*: Total-IKK-*β*
TNFRI: TNF receptor I
TNFRII: TNF receptor II
mAb: Monoclonal antibody
NF-*κ*B: Nuclear factor kappa-B
BM: Bone marrow
MNCs: Mononuclear cells
SDF-1*α*: Stromal cell derived factor-1*α*
CXCR4: C-X-C chemokine receptor type 4
VCAM-1: Vascular cell adhesion molecule 1
CFSE: 5(6)-Carboxyfluorescein diacetate succinimidyl ester
SLEDAI: Systemic lupus erythematosus disease activity index
EDTA: Ethylene diamine tetraacetic acid
TPCA-1: 2-[(Aminocarbonyl)amino]-5-(4-fluorophenyl)-3-thiophenecarboxamide
MAPK: Mitogen-activated-protein-kinase
ERK: Extracellular signal-regulated kinase
JNKs: c-JUN N-terminal kinase
PI3K: Phosphoinositide-3-kinase
L-DMEM: Low glucose Dulbecco Modified Eagle Medium
FBS: Fetal bovine serum
OD: Optical density
RT-PCR: Real-time polymerase chain reaction
ELISA: Enzyme-linked immunosorbent assay
SDS-PAGE: Sodium dodecyl sulfate polyacrylamide gel electrophoresis
PVDF: Polyvinylidene difluoride
ICAM-1: Intercellular adhesion molecule-1
MMP-9: Matrix metalloproteinases
PDGF: Platelet-derived growth factor
HGF: Hepatocyte growth factor
MMP-2: Matrix metalloproteinase 2
VEGF: Vascular endothelial growth factor
ANA: Anti-nuclear antibody
anti-dsDNA: Anti-double strand DNA antibody
anti-RNP: Anti-ribonuclear protein antibody
anti-SM: Anti-Smith antibody
Pred: Prednisone
CYC: Cyclophosphamide
HCQ: Hydroxychloroquine
MMF: Mycophenolate mofetil
TNFRAIL: Tumor necrosis factor-related apoptosis-inducing ligand
IFN-*γ*: Interferon-*γ*
PDGF: Platelet-derived growth factor
IL-6: Interlukin-6.
