# Supplementary material for: Association of TNF-α with Impaired Migration Capacity of Mesenchymal Stem Cells in Patients with Systemic Lupus Erythematosus
Source: J Immunol Res. 2014 Nov 24;2014:169082. doi: 10.1155/2014/169082 (PMC4265382; doi:10.1155/2014/169082)
Supplement: Supplementary file 1 — Supplementary Figure 1: Associations of the migration rate with clinical manifestations: a. SLEDAI score, b. white blood cells, c. hemoglobin, d. platelet, e. urine protein, f. serum creatinine, g. complement 3, h. complement 4, i. ESR, j. arthralgia, k. febrile, l. vasculitis, m. pneumonia, n. serositis. Supplementary Figure 2: Associations of the migration rate with various treatment drugs. a, b. BMSCs from patients taking high doses of steroids (a) or HCQ (b) tended to have decreased migration rate. c,d. MMF (c) or CYC (d) treatment do not seem to have effect on SLE BMSCs migration rate. [file 169082.f1.pdf]

Supplementary Figure 1.

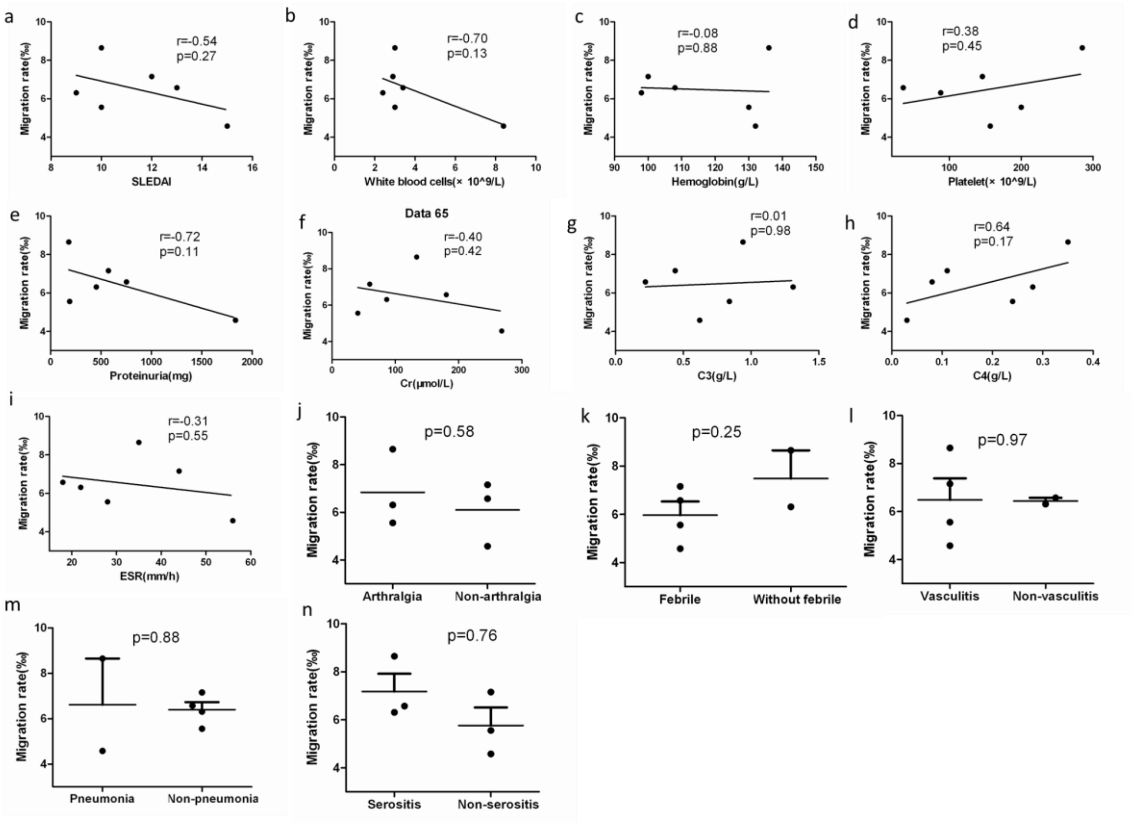

Supplementary Figure 1. Associations of the migration rate with clinical manifestations: a. SLEDAI score, b. white blood cells, c. hemoglobin, d. platelet, e. urine protein, f. serum creatinine, g. complement 3, h. complement 4, i. ESR, j. arthralgia, k. febrile, l. vasculitis, m. pneumonia, n. serositis.

Supplementary Figure 2.

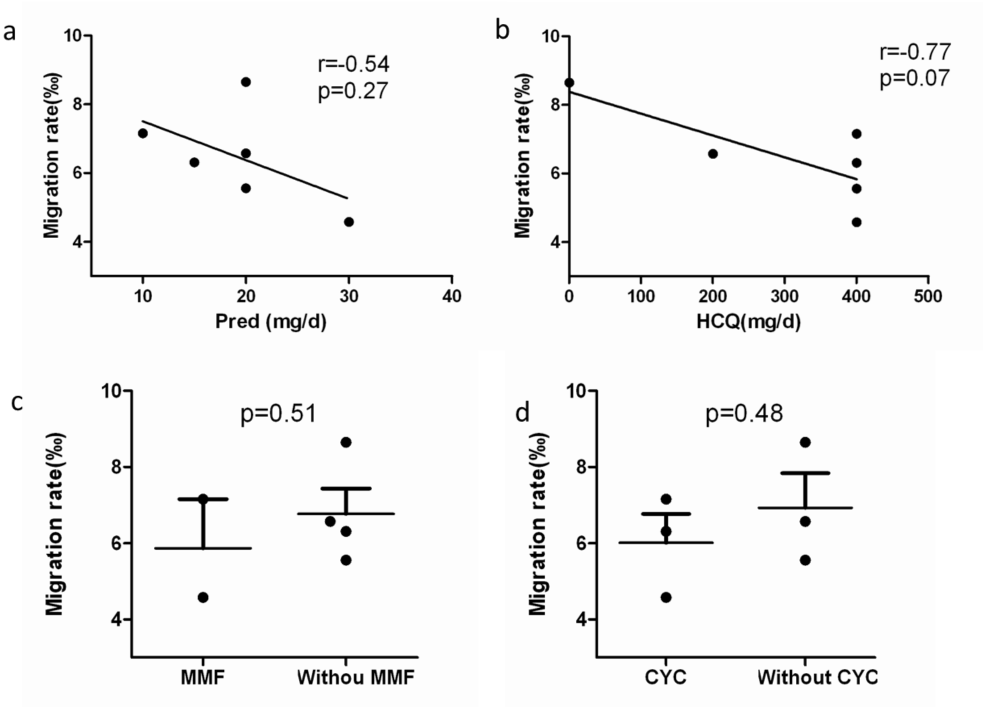

Supplementary Figure 2. Associations of the migration rate with various treatment drugs. a. Pred, b. HCQ, c. MMF, d. CYC.
